# Identification of markers associated with global changes in DNA methylation regulation in cancers

**DOI:** 10.1186/1471-2105-13-S13-S7

**Published:** 2012-08-24

**Authors:** Peng Qiu, Li Zhang

**Affiliations:** 1 Department of Bioinformatics and Computational Biology, The University of Texas MD Anderson Cancer Center, Houston, TX, USA

## Abstract

DNA methylation exhibits different patterns in different cancers. DNA methylation rates at different genomic loci appear to be highly correlated in some samples but not in others. We call such phenomena conditional concordant relationships (CCRs). In this study, we explored DNA methylation patterns in 12 common cancers using data of 2434 patient samples collected by The Cancer Genome Atlas project. We developed an exploratory method to characterize CCRs in the methylation data and identified the 200 gene markers whose on-and-off statuses in DNA methylation are most significantly associated with drastic changes in CCRs throughout the genome. Clustering analysis of the methylation data of the 200 markers showed that they are tightly associated with cancer subtypes. We also generated a library of the significant CCRs that may be of interest to future studies of the regulation network of DNA methylation in cancer.

## Introduction

DNA methylation plays an important role in carcinogenesis and cancer progression through hypermethylation to turn off the expression of tumor suppressors and hypothmethylation to activate the expression of oncogenes [1]. Genomic analyses of DNA methylation using microarrays and next generation sequencing technologies have shown that various forms of neoplasia and cancers are associated with massive changes in DNA methylation [2,3]. Such changes are often distinctive depending on the subtype of cancer [4,5]. DNA methylation in cells is apparently regulated by a large, intricate network. However, although a large number of genomic network studies have focused on data regarding gene expression, protein-protein interactions, and protein-DNA/RNA interactions [6,7], little has been done to incorporate DNA methylation data to study the underlying regulatory network.

In general, relationships that link different genes at DNA, RNA, protein, and metabolite levels strongly depend on the specific context, such as cell type, subcellular location and time of the biological processes. A number of methods have been developed to uncover context-dependent relationships using gene expression data. For example, the liquid association model was developed to identify mediator genes that can modulate coexpression of other pairs of genes [8]. A few other similar models have been proposed to describe three-way relationships among genes [9-11]. Cancer type dependent coexpression patterns have been reported previously [12,13]. The MINDy algorithm used conditional mutual information to identify modulators that strongly affect the concerted activities of transcription factors and their targets, and found novel modulators of MYC function in B cells [14].

In this study, we focused on the dynamic nature of concordant relationships between the methylation status of genes, using a large DNA methylation dataset of 2434 samples across 12 cancer types generated by The Cancer Genome Atlas (TCGA) project. We observed that many gene pairs showed dramatic changes in methylation patterns in different cancers. We call such phenomena conditional concordant relationships (CCRs). CCRs are commonly observed in cancer. For example, Hess et al. found that methylation of the ESR1 promoter is strongly predictive of the concurrent methylation of a group of tumor suppressors in acute myeloid leukemia, and is associated with clinical outcome [15]. Carvalho et al. found that the concurrent methylation of a group of cancer-related genes is associated with the microsatellite instability phenotype [16]. We are particularly interested in finding marker genes that have the following property: depending on the methylation status of the marker, the patient samples can be dichotomized into two groups, and the gene-gene correlation matrices derived from the methylation data of the two groups are drastically different. Such markers are likely to be associated with global changes in methylation correlation patterns. This concept of the methylation markers resembles the modulator in three-way gene expression studies [9]. We have developed a method to identify such markers. We demonstrate the utility of our approach to study CCRs, classify cancer subtypes, and explore the patterns of DNA methylation in cancer.

## Results

### Genomic patterns of DNA methylation in cancers

**Figure 1:**
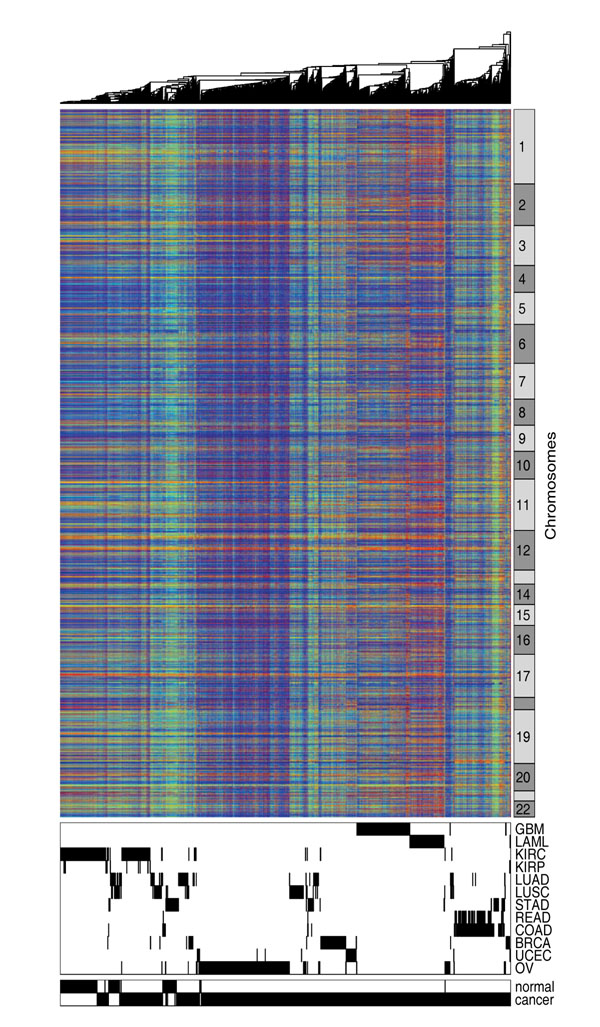
**Clustering of whole genome methylation pattern**. Hierarchical clustering is performed using the whole genome methylation data observed in 2434 samples across 12 common cancers.

To show the overall pattern of DNA methylation in cancers, we downloaded methylation data of 2434 samples across 12 cancer types from the TCGA data portal [17] and performed hierarchical clustering analysis. Table 1 shows the sample size for each of the 12 cancer types. This dataset contains 27,578 probes interrogating proximal promoter regions of 14,475 genes in the human genome. The methylation status of many probes showed small variances and therefore does not contribute to the clustering analysis. We hence removed the non-changing probes and kept ~9000 probes that have the highest variance across samples. We also removed probes on the X and Y chromosomes because their methylation rates mainly reflected gender difference rather than disease or tissue differences. Figure 1 shows the cluster diagram generated from the 9000 probes across 2434 samples, with each row representing one probe and each column representing one sample. The bottom panel shows the tissue type and normal-cancer status of the samples. It can be observed that the samples were mostly organized by tissue type, with some noticeable outliers. GBM, LAML, OV, BRAC and UCEC samples formed their own clusters. READ and COAD samples were grouped together. The kidney cancer samples and the normal kidney samples were clustered close to each other. The normal and cancer samples of LUAD and LUSC were mixed with each other but scattered across the clustering diagram. The majority of the lung samples appeared to be similar to KIRC. The STAD samples formed three groups. A subset of the STAD cancer samples were clustered with READ and COAD, while the remaining STAD cancer samples were clustered with lung cancer samples. The STAD normal samples were similar to another group of lung cancer samples.

**Table 1:** Cancer type and sample size of TCGA methylation data

Cancer Type	Sample size	
	cancer	normal
GBM - Glioblastoma multiforme	291	0
LAML - Acute myeloid leukemia	188	0
KIRC - Kidney renal clear cell carcinoma	219	199
KIRP - Kidney renal papillary cell carcinoma	16	5
LUAD - Lung adenocarcinoma	128	24
LUSC - Lung squamous cell carcinoma	134	27
STAD - Stomach adenocarcinoma	82	59
READ - Rectum adenocarcinoma	70	1
COAD - Colon adenocarcinoma	168	15
BRCA - Breast invasive carcinoma	186	0
UCEC - Uterine corpus endometrioid carcinoma	70	0
OV - Ovarian serous cystadenocarcinoma	542	10

**Figure 2:**
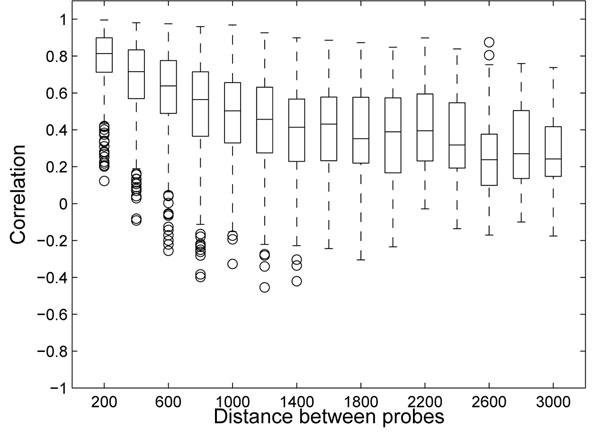
**Correlation of methylation and genomic distance**. Correlation of methylation depends on distance between probed positions on the chromosomes. The box plots show the distribution of correlation (y-axis) between two neighboring probes depending on the distance between the interrogated loci.

Since the rows in Figure 1 were ordered according to chromosomal location, the horizontal stripes indicate that methylation patterns are similar for the probes that are close to each other on the chromosomes. In Figure 2, we show that the correlation of methylation rates between two probes is related to the distance between the genomic loci that the probes interrogate. The correlation was high (> 0.7 in 75% of the case) for probes within 200 base pairs (bps) of one another. As we examined probe pairs with larger distances, the correlation diminished and decreased to a baseline level beyond 2000 bps.

**Figure 3:**
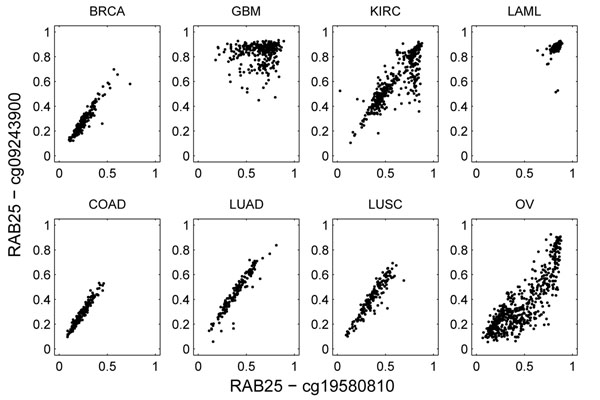
**Example of cancer-dependent methylation correlation.** The methylation data of two probes in the promoter region of RAB25 is used to derive the plots. Each plot shows the correlations of methylation level between the two probes in one cancer type.

We noticed that the pairwise relationships of methylation rates between two genomic loci can strongly depend on cancer type. For example, Figure 3 shows the methylation data for two probes that both interrogate the promoter region of gene RAB25 on chromosome 1. The two probe loci are 338 bps apart. In COAD, BRCA, LUAD, and LUSC, the methylation rates between the probes were highly correlated (> 0.9). In LAML, both loci were always fully methylated. In KIRC and OV, the linear correlation was somewhat distorted. In GBM, however, the correlation appeared to be zero, which is strikingly different from the other cancers. We consider this to be a clear example of CCRs.

### Identifying the markers associated with global changes in gene-gene correlations

**Figure 4:**
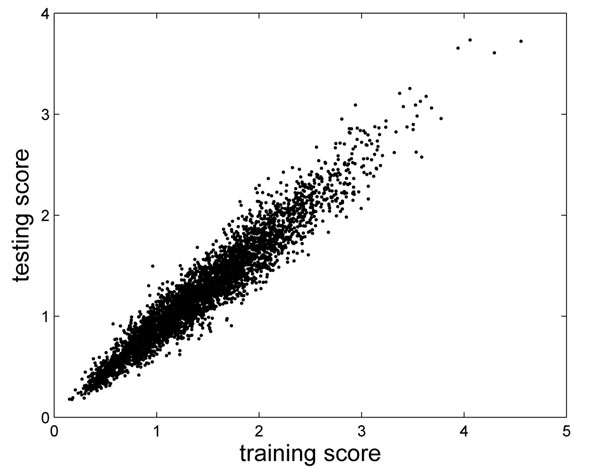
**Scores that describe CCR-association.**Each of the 9000 high-variance probes is evaluated and scored on whether it is associated to CCR. Two sets of scores are derived from the training and testing data separately, and the two sets of scores show high correlation.

To systematically evaluate CCRs, we searched for marker probes associated with a large number of them. We randomly selected 1500 samples as the training set, and the remaining samples were reserved as the testing set. Based on the training samples, we selected ~9000 high-variance probes, derived scores to evaluate each probe’s association with CCRs, and rank-ordered the probes (see Methods). These high-variance probes were also scored based on the testing set. Figure 4 shows that the scores obtained in the training set were highly correlated with those derived from the testing set. This procedure was repeated multiple times, and the results appeared to be nearly identical (details not shown), suggesting that the top-ranked probes and their scores were robust.

**Figure 5:**
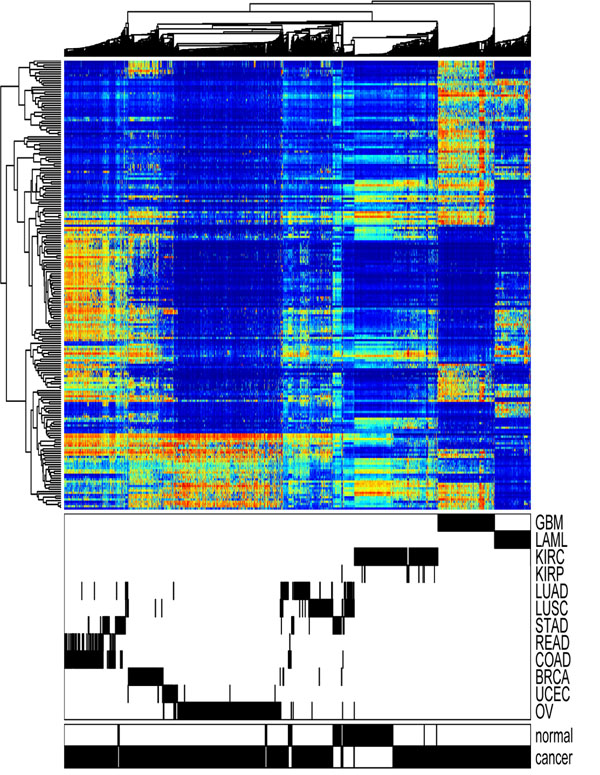
**Clustering of top-200 CCR-associated probes**. Hierarchical clustering of the 2434 cancer samples is performed using 200 top CCR-associated probes.

We performed clustering analysis on all 2434 samples based on the top 200 probes selected from the training set. As shown in Figure 5, the top 200 CCR-associated probes were able to separate cancer types. Similar to the previous analysis in Figure 1 based on 9000 high-variance probes, the top CCR-associated probes defined distinct clusters for GBM, LAML, OV, BRAC, and UCEC, respectively. READ and COAD samples were grouped into one cluster. The major difference was the clustering of the lung samples. Based on the CCR-associated probes, the two subtypes of lung samples (LUAD and LUSC) formed one tight cluster. Normal lung samples were grouped with KIRCs in the previous analysis, but the CCR-associated probes highlighted the difference between them.

For most cancer types where normal and cancerous samples were both available, the cancerous and corresponding normal samples were clustered close to each other. This observation suggests that the difference in methylation across different tissue types is larger than cancer-induced methylation changes. The only exception in this dataset was STAD. In Figure 5, we observed that the STAD normal samples were more similar to the lung samples, whereas the STAD cancer samples were more similar to the COAD and READ samples. This observation indicates that methylation might play a major role in stomach adenocarcinoma.

**Figure 6:**
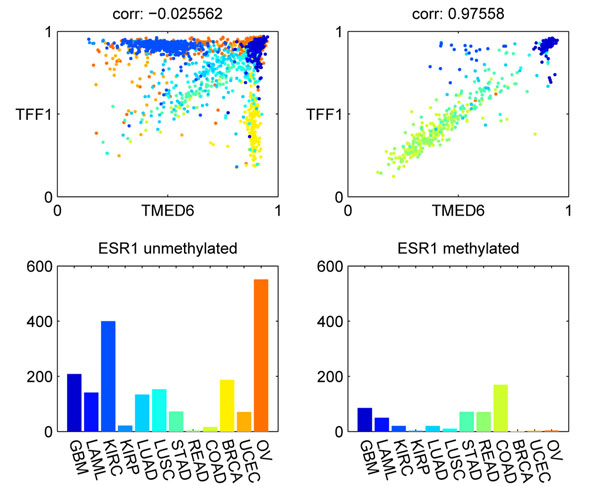
**Example of CCR-associated marker ESR1**. The methylation status of ESR1 affects the correlation between TMED6 and TTF1. The left panels show the correlation between TMED6 and TTF1, and cancer type distribution for samples in which ESR1 is unmethylated. The right panels correspond to the samples with ESR1 methylated.

Our analysis found ESR1 to be one of the most significant CCR-associated markers. Figure 6 shows an example of a CCR associated with ESR1. When ESR1 was methylated, the correlation between TMED6 and TFF1 was high. The correlation was disrupted in when ESR1 was unmethylated. In BRCA samples, TMED6 was always methylated, while TFF1 could be either methylated or unmethylated. This was in sharp contrast to the high correlation between TMED6 and TFF1 in COAD samples. ESR1 is known to play a very important role in cancer, and previous research found that ESR1 methylation is associated with concurrent methylation of a group of tumor suppressors [15]. TFF1 is an ESR1 regulated protein, and it has been found to enhance cell migration and oncogenicity in breast cancers [18,19]. TMED6, transmembrane emp24 protein transport domain containing 6, has been found to be differentially expressed in different grades of gastric cancer [20], but no direct evidence has been found to implicate its role in cancer.

**Figure 7:**
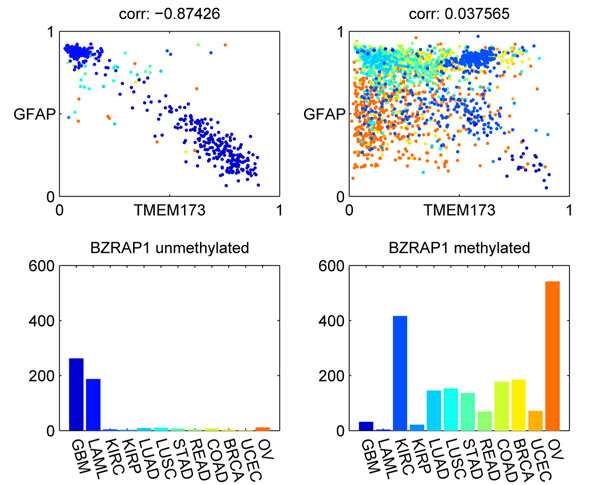
**Example of CCR-associated marker BZRAP1**. The methylation status of BZRAP1 affects the correlation between TMEM173 and GFAP. The left panels show the correlation between TMEM173 and GFAP when BZRAP1 is unmethylated. The right panels correspond to the samples with BZRAP1 methylated.

Figure 7 shows another example of a CCR-associated marker, BZRAP1. This example is interesting because there was a strong negative correlation between GFAP and TMEM173 in GBM samples, where BZRAP1 was unmethylated. It is not clear how these three genes are related. BZRAP1 is an autism risk gene. TMEM173 expression can activate the NF-*κ*B signaling pathway [21]. GFAP is a cell specific marker of astrocytes in the brain and is regulated by NF-*κ*B [22]. However, the functions of these genes do not provide a clear clue about the observed negative correlation between TMEM173 and GFAP.

### CCR-associated markers recovered GBM subtypes

**Figure 8:**
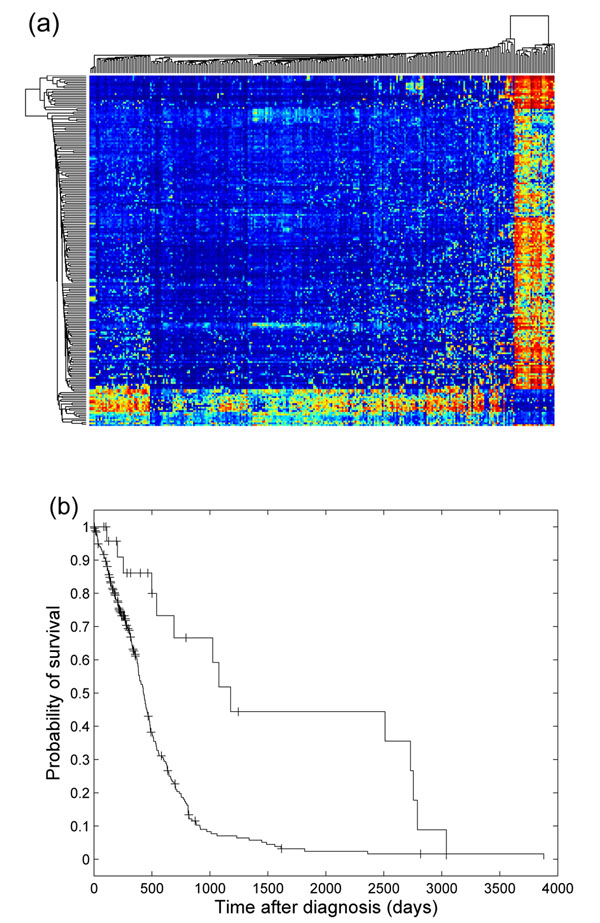
**CCR-associated markers and GBM subtypes**. (a) Clustering and diagram of 291 GBM samples based on top 200 CCR-associated probes. (b) Kaplan-Meier plot of the survival data of the two GBM subgroups observed in (a). The survival of the two groups showed significant differences (logrank test, pvalue 10^–5^).

In the previous section, we showed that when applied to all samples containing multiple tissue types, the top CCR-associated markers were able to distinguish among tissue types. A natural next step was to focus on one cancer type, and examine whether the CCR-associated markers could identify cancer subtypes. We focused on the 291 GBM samples, selected ~9000 high variance probes, scored each probe’s association with CCRs, rank-ordered the probes, and used the top 200 probes to perform clustering analysis. Figure 8 (a) shows the clustering diagram of the 291 GBM samples based on the top 200 CCR-associated probes. We observed that the GBM samples were divided into two groups. The clinical outcome of the smaller group was significantly better than that of the bigger group, as shown in Figure 8 (b). The smaller GBM sample group with better survival was previously reported [23]. This group of samples carry a CpG island methylator phenotype, which is associated with better survival and low-grade gliomas. In Noushmehr et al. [23], clustering analysis was performed on 1500 high-variance probes, and discovered three GBM subtypes. One of the three was the smaller sample group we show in Figure 8 (a). The remaining two corresponded to the bigger group in our analysis, but there was no significant evidence for biological and clinical differences between the two remaining groups in the previous study.

## Discussion

We have described an approach to explore complex patterns observed in DNA methylation data. We identified CCRs and markers associated with global changes in methylation correlation in different cancers. Expectedly, when the identified markers were used for clustering analysis, the clustering diagram largely coincided with cancer types, since distinct methylation patterns exist in different tissue types. We demonstrated that our approach can be used to uncover tissue types and subtypes of cancer. In this sense, our method is similar to feature selection and unsupervised clustering.

However, there are also important distinctions. In clustering methods, the common approach is to divide samples into groups so that within-group variation is small and between-group variation is large. In contrast, our method seeks markers that define two sample groups whose within-group correlation patterns differ. We do not require within-group variation to be small.

It should be noted that the associations between the markers and CCRs shown in this study are statistical associations identified from the data. The markers are not necessarily the causative agents that drive the changes in the correlations. Nevertheless, the markers provide candidates and useful information to identify the underlying causative agents. We believe that the main utility of our approach is to facilitate a systematic assessment of CCRs, which could be useful toward a better understanding of DNA methylation regulation in cancer.

The current study was limited to methylation data only. However, data from multiple platforms measuring gene expression, microRNA expression, DNA copy number, and somatic mutations can all be evaluated as candidate markers that affect CCRs in DNA methylation. Integrating data from multiple platforms will be increasingly powerful as more data are accumulated in the TCGA project.

## Method

### Data and preprocessing

In this study, we focused on the DNA methylation data provided by TCGA (http://tcga-data.nci.nih.gov/tcga/tcgaHome2.jsp). Genome-wide methylation measurements of 2434 samples were available, spanning across 12 cancer types. The data were generated using the IIllumina Infinium Human DNA Methylation27 array platform, which interrogates the methylation status of 27,578 CpG sites for each sample. The data is available at http://odin.mdacc.tmc.edu/~pqiu/projects/TCGAMethData/index.htm.

We used the level 3 methylation data defined by TCGA, which is the ratio of *M_i _*/(*U_i_* + *M_i_*) for each CpG site *i*. *M_i_* represents the methylated probe intensity of CpG site *i*, while *U_i_* is the unmethylated probe intensity. Therefore, the numerical range of the data is between 0 and 1. 0 means unmethylated, and 1 means completely methylated. The data contain null entries, which correspond to probes that overlap with known single nucleotide polymorphisms (SNPs) or other genomic variations, and probes whose signal intensities are lower than the background.

In our analysis, we filtered out probes with many null entries (number of nulls more than 1% of the sample size) and probes with small standard deviation (*SD* < 0.1). Roughly 9000 probes survived these two filtering criteria and were considered in the analysis of CCRs.

### Dichotomize samples based on methylation

Although DNA methylation is a reversible process and methylated CpG sites may not be completely methylated, methylation data appear to be bimodal in general. By thresholding the ratio *M_i _* /(*U_i_* + *M_i_*) (i.e., nominal threshold 0.2), we can use probe *i* to divide samples into two groups. The status of CpG site *i* in one group is unmethylated, whereas CpG site *i* in the other group is methylated. If the methylation correlation patterns in the two sample groups are quite different, the CpG site *i* is likely to be related to the global changes in methylation regulation.

### Clustering

Before calculating the changes in methylation correlation, clustering is performed to find modules of highly correlated probes. The purpose of this step is to reduce computational complexity. The pairwise correlations between modules can be used as surrogates of the pairwise correlations between individual probes.

We use a variation of the agglomerative clustering algorithm [24,25]. This algorithm requires a user-specified threshold for cluster coherence, defined as the average Pearson correlation between each probe in the cluster and the cluster mean. This parameter determines the quality of the resulting clusters (the default setting is 0.7). At the beginning of the first iteration of the agglomerative algorithm, each probe forms its own cluster. One probe is randomly chosen and merged with its nearest neighbor as defined by Pearson correlation and average linkage, and these two probes become unavailable in the current iteration. Then, another probe is randomly chosen from the remaining ones and merged with its nearest neighbor, if the nearest neighbor is still available. Again, the chosen probe and its nearest neighbor become unavailable in the current iteration. If a merge results in a cluster whose coherence is below the user-specified threshold, the merge is rejected. After all the probes become unavailable, the first iteration ends and the number of clusters is reduced by approximately half. The same procedure is repeated in the second iteration to further reduce the number of clusters. The iterative process continues until all merges in a particular iteration are rejected.

This algorithm guarantees that the quality of all the resulting probe clusters is higher than the user-specified threshold. The average of each cluster can be viewed as a meta-probe that summarizes the average methylation status of the cluster of correlated probes.

### Identify CCR-associated switch-like probes

To identify CCR-associated probes, we used the training samples to filter for roughly 9000 probes that had small number of null entries and high standard deviation. These probes were considered as candidates to be evaluated. We also performed the above agglomerative algorithm using the training set to cluster probes into modules that contained highly correlated probes, and we represented each module by the mean methylation profile of probes in that module.

For each candidate probe, we evaluated whether its on-off status affected methylation correlation globally. We dichotomized the training samples into two groups (i.e., threshold = 0.2), computed the module-module correlation matrices for the two sample groups separately, performed z-transform, and summarized the difference between the two correlation matrices into one scalar score (*s* = ∑*_i_*_,_*_j_* |*z*_1_(*i*, *j*) – *z*_2_(*i*, *j*)|). If a candidate probe resulted in an extremely unbalanced split (i.e., the smaller sample group contained less than 15% of samples), this candidate probe was not scored, because correlation computed from on a small number of samples may not be accurate and reliable. The candidate probes were rank-ordered according to their scores, where the methylation status of top ranking probes were associated with large changes in methylation correlation.

## Competing interests

The authors declare that they have no competing interests.

## Authors' contributions

PQ and LZ conceived of the study, developed the method and wrote the manuscript.
